# Visual Outcomes of a Non-Diffractive Extended Depth-of-Focus Intraocular Lens in Patients with Early-Stage Age-Related Macular Degeneration

**DOI:** 10.3390/jcm14175953

**Published:** 2025-08-23

**Authors:** Emilio Dorronzoro-Ramirez, Miguel Angel Sanchez-Tena, Cristina Alvarez-Peregrina, Jose Miguel Cardenas Rebollo, Dayan Flores Cervantes, Celia Sánchez-Ramos

**Affiliations:** 1 Faculty of Medicine, CEU-San-Pablo University, 28925 Madrid, Spain; emilio.dorronzoro@usp.ceu.es (E.D.-R.); cardenas@ceu.es (J.M.C.R.); 2 Hospital Universitario Sanitas La Moraleja, 28050 Madrid, Spain; mdflores@sanitas.es; 3 Faculty of Optics and Optometry, Universidad Complutense de Madrid, 28037 Madrid, Spain; cristina_alvarez@ucm.es (C.A.-P.);; 4 ISEC LISBOA-Instituto Superior de Educação e Ciências, 1750-179 Lisbon, Portugal; 5 Vision and Ophthalmology Research Group, Universidad Complutense de Madrid, 28037 Madrid, Spain

**Keywords:** extended depth of focus IOL, non-diffractive IOL, age-related macular degeneration, cataract surgery, visual acuity, LuxSmart, Tecnis Eyhance, patient-reported outcomes

## Abstract

**Background/Objectives**: Age-related macular degeneration (AMD) is a leading cause of visual impairment in older adults and often coexists with cataracts. The indication of presbyopia-correcting intraocular lenses (IOLs) in these patients remains controversial. This study aimed to evaluate the clinical performance of a non-diffractive extended depth-of-focus (EDOF) IOL (LuxSmart™) compared to a monofocal plus IOL (Tecnis Eyhance™) in cataract patients with early-stage dry AMD. **Methods**: In this prospective observational study, 41 patients with early-stage AMD underwent bilateral cataract surgery with either LuxSmart™ or Tecnis Eyhance™ IOL implantation, targeting postoperative emmetropia. The eye selected for analysis was the first eye scheduled for surgery. Preoperative and postoperative evaluations included high and low-contrast distance visual acuity, intermediate and near visual acuity, defocus curves, ocular light scatter (halometry), and quality of life assessment (NEI VFQ-25). Postoperative biometric accuracy and refractive outcomes were also analyzed. **Results**: Both IOLs showed high refractive accuracy, with 100% of eyes within ±0.50 D of target. Postoperative uncorrected distance visual acuity was 0.10 ± 0.06 LogMAR for Eyhance and 0.07 ± 0.02 for LuxSmart (*p* = 0.06). Low contrast VA at 20% was 0.22 ± 0.11 (Eyhance) and 0.26 ± 0.16 (LuxSmart) (*p* = 0.49). Depth of focus was approximately 1.75 D for both lenses. Light scatter (LDI) improved postoperatively in both groups with no significant differences (*p* = 0.54). VFQ-25 scores showed improvement in daily activities, though no changes were observed in driving or mental health domains. **Conclusions**: Both lenses are safe and effective options for early AMD patients undergoing cataract surgery, providing good functional vision at multiple distances

## 1. Introduction

Age-related macular degeneration (AMD) is one of the leading causes of visual impairment in people over the age of 55. This condition, often coinciding with the presence of cataracts, poses a significant challenge for cataract surgery, especially in patients seeking greater independence from glasses after surgery, as it can influence the choice of intraocular lens to be implanted. According to the Functional Vision Working Group of the European Society of Cataract and Refractive Surgeons (ESCRS), Europeans over the age of 55 spend an average of six hours a day on leisure activities that require good intermediate vision, such as using electronic devices, reading, and socializing [1].

The use of presbyopia-correcting intraocular lenses (IOLs) in patients with AMD has long been a topic of debate within the field of cataract surgery [2,3]. These lenses, designed to provide visual independence from optical correction at different distances, often relied on diffractive optics, which can reduce the amount of light reaching the retina and affect visual performance in low-light conditions [4]. These effects may further compromise the already diminished retinal function in AMD patients. Recently, a new group of extended depth of focus (EDOF) lenses that do not use diffractive optics has been developed, offering a promising alternative for patients with early-stage AMD. These lenses employ refractive optics designed to extend the depth of focus without splitting light into multiple focal points. This design avoids light loss and contrast degradation typically associated with diffractive IOLs. They provide a greater range of vision without the need for additional optical correction for intermediate vision, representing a viable option for patients requiring cataract surgery [5,6].

The main objective of this study is to evaluate the visual performance of the non-diffractive EDOF LuxSmart™ lens (Bausch & Lomb GmbH, Berlin, Germany) in cataract-operated patients with early-stage AMD, comparing it with the monofocal plus Tecnis Eyhance™ lens (ICB00, Johnson & Johnson Surgical Vision, Inc., Santa Ana, CA).

Therefore, the study aims to analyze visual acuities at different distances (far, intermediate, and near) under high and low-contrast conditions, quantify light scatter within the eye, evaluate defocus curves, and assess the quality of life in patients with early-stage AMD undergoing cataract surgery.

## 2. Materials and Methods

This prospective observational study included patients who underwent bilateral implantation of either the monofocal plus Tecnis Eyhance™ intraocular lens (Lens A) or the non-diffractive extended depth-of-focus LuxSmart™ intraocular lens (Lens B) following femtosecond laser-assisted phacoemulsification (FLACS). All procedures aimed for postoperative emmetropia.

The study analyzed data from patients diagnosed with cataracts using slit-lamp biomicroscopy with the Yuratec RT 1000 (Carl Zeiss, Jena, Germany).

To diagnose early dry AMD, Optical Coherence Tomography (OCT) with the Dri OCT Triton^®^ instrument (Topcon, Itabashi, Japan) was used to detect the presence of AMD and classify it using the 3D retinal map acquisition protocol. The severity of AMD was graded according to the classification system from the Age-Related Eye Disease Study (AREDS) [7]. Only eyes with AREDS category 2 (early-stage dry AMD) were included.

Category 1: No AMD (small drusen < 63 µm)

Category 2: Early or initial AMD (many small drusen or few medium drusen 63–125 µm)

Category 3: Intermediate AMD (many medium drusen or 1 large druse > 125 µm in one or both eyes, or non-central geographic atrophy)

Category 4: Advanced AMD (central geographic atrophy or choroidal neovascularization)

Inclusion criteria were uncomplicated cataract surgery, clear posterior capsule and implanted lens, absence of pupillary anomalies, and the ability to read and understand questionnaires. Exclusion criteria included suspected glaucoma, optic neuropathies, diabetic retinopathy or vasculopathy, advanced AMD, presence of maculopathy other than AMD, traumatic or metabolic cataract, pseudoexfoliation, severe dry eye, irregular corneal astigmatism, extreme pupil size (photopic value <2.00 mm or >4.00 mm), previous corneal or intraocular refractive surgery, corneal anomalies, IOL dislocation, posterior capsule opacification, corneal astigmatism >3.00 diopters (D), or any vitreous or retinal disease.

Regarding ethical considerations, this research followed the principles of the Declaration of Helsinki and was approved by the Ethics Committee of the Hospital Clínico San Carlos in Madrid (code 23/064-E).

### 2.1. Intraocular Lens

All patients were randomly assigned to receive either the Tecnis Eyhance™ IOL (ICB00, Johnson & Johnson Surgical Vision, Inc., Santa Ana, CA, USA) or the LuxSmart™ IOL (Bausch & Lomb GmbH, Berlin, Germany). Regarding the eye selected for analysis, we chose the first eye scheduled for surgery in each patient to maintain consistency. As for the allocation of intraocular lenses, we used a computer-generated randomization sequence through the Sealed Envelope service (https://www.sealedenvelope.com/simple-randomiser/v1/lists -accessed on 30 January 2025), which assigned patients to either the LuxSmart™ or Tecnis Eyhance™ group prior to surgery.

Tecnis Eyhance™ (ICB00, Johnson & Johnson Surgical Vision, Inc., Santa Ana, CA, USA) is a monofocal plus posterior chamber intraocular lens, biconvex, made of hydrophobic acrylic material. It has a total diameter of 13 mm and an optical zone diameter of 6 mm. Its posterior surface is spherical, while the anterior surface is aspherical. The main advantage of this design is its wide depth of focus due to its asphericity increasing from the periphery to the center, resulting in an equivalent of +1.25 diopters that allows improved intermediate vision (hence the term monofocal plus or mono EDOF) compared to other standard aspherical monofocal lenses [8]. The material has an Abbe number of 55 and a refractive index of 1.47. For a pupil of 6 mm, which equals 5.15 mm in the lens plane, it produces 0.27 µms of fourth-order negative spherical aberration to compensate for the positive spherical aberration of the cornea.

LuxSmart™ (Bausch & Lomb GmbH, Berlin, Germany) is engineered with Pure Refractive Optics (PRO) technology. It is a posterior chamber intraocular lens, one-piece, made of hydrophobic acrylic material. It has a refractive surface along the entire optical zone of 6 mm without a diffractive profile and a total diameter of 11 mm. The center, with an extended depth of focus of 2 mm, is achieved through a combination of fourth and sixth-order spherical aberrations of opposite signs, designed to increase subjective depth of field up to +1.75D (Pure Refractive Optics design to provide an extended range of vision, PRO). It has a transition zone to smoothly decrease optical vergence from the center to the periphery, designed to manage spherical aberrations. It allows controlling the trajectory of light rays and ensures they do not deviate outside the range of vision. The periphery has an aspherical monofocal design, free of aberrations. It has an Abbe number of 43 and a refractive index of 1.54.

### 2.2. Clinical Procedure

Before surgery, patients underwent a complete ophthalmological examination, which included measuring distance visual acuity (VA) with their best optical correction. The diagnosis and classification of cataracts were performed using slit-lamp and following the Lens Opacities Classification System III (LOCS III) [9]. Ocular biometry was conducted using the IOLMaster 700 (Carl Zeiss Meditech, Jena, Germany), which allowed for the measurement of axial length, keratometry, white-to-white distance, anterior chamber depth, lens thickness, and pupil diameter. These biometric parameters were used to calculate the intraocular lens power with the Barrett True-K formula, targeting emmetropia in all cases.

Additionally, objective refraction was obtained with the Visuref^®^ 150 autorefractometer (Carl Zeiss, Jena, Germany), high and low contrast visual acuity was assessed with the Colenbrander Mixed Contrast Chart backlit screen calibrated to measure at 1 m, and defocus curves. Ocular light scatter evaluation was conducted using the Halo v.1.0 software created by the Vision Sciences and Applications Laboratory of the University of Granada, Spain. The Halo Software User Manual can be found in the Supplementary Materials. Corneal topography was assessed using the Pentacam HR^®^ topographer (Oculus Optikgeräte GmbH, Wetzlar, Germany), which evaluated high-order aberration (HOA) and spherical aberration (z40) using a standardized pupil diameter of 6.0 mm, as provided by the Pentacam HR^®^ system. Endothelial cell counts were measured using the SP-3000P^®^ instrument (Topcon, Japan), and intraocular pressure was measured using the CT-80^®^ air tonometer (Topcon, Japan).

### 2.3. Surgical Procedure

All patients underwent cataract surgery using femtosecond laser-assisted phacoemulsification (FLACS), following standard clinical procedures. The surgeries were performed by the same experienced surgeon under topical anesthesia through a microincision of 2.0 to 2.2 mm made with a pre-calibrated knife.

For cataract surgery, the Victus Technolas™ femtosecond laser (Bausch & Lomb GmbH, Berlin, Germany) and the Stellaris Elite™ phacoemulsifier (Bausch & Lomb GmbH, Berlin, Germany) were used. A Zeiss Lumera 700 operating microscope (Carl Zeiss Meditec, Jena, Germany) was used for all intraocular procedures. A viscoelastic Combivisc, marketed by Zeiss (Carl Zeiss Meditec, Jena, Germany), was chosen for its combination of dispersive viscoelastic (based on sodium hyaluronate at a concentration of 30 mg/mL) and cohesive viscoelastic (based on sodium hyaluronate at a concentration of 15 mg/mL). For the removal of lens fragments, the BL5113 pressurized infusion cassette of the Stellaris Elite™ (Bausch & Lomb GmbH, Berlin, Germany) was used, providing greater stability in the anterior chamber of the eye. The parameters during the fragment removal phase consisted of an intraocular pressure of 35 mmHg, ultrasound power at 35%, and a microflow tip, all designed to enhance the performance of phacoemulsification.

### 2.4. Postoperative Evaluation

**Figure 1:**
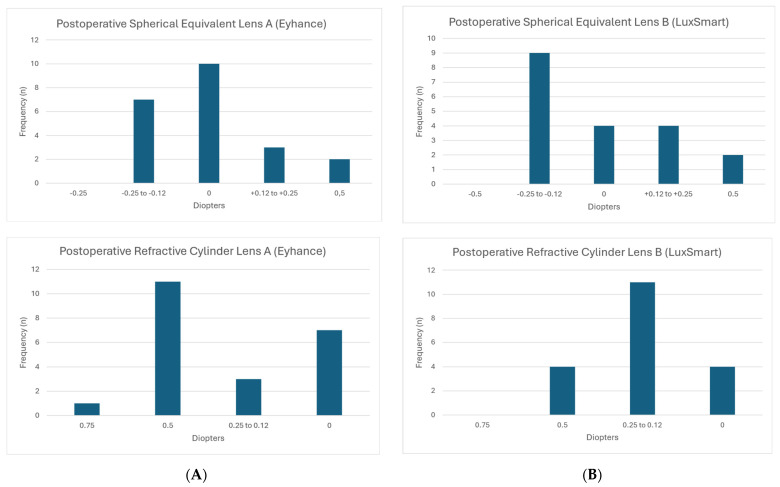
(**A**) Distribution of the spherical equivalent and postoperative refractive cylinders in diopters (D) for Lens A (Eyhance). (**B**) Distribution of the spherical equivalent and postoperative refractive cylinders in diopters (D) for Lens B (LuxSmart).

After cataract surgery, patients underwent postoperative follow-up visits to evaluate their visual and ocular status one month after surgery. The accuracy of the biometric calculation was verified by determining the subjective refraction and calculating the postoperative spherical equivalent and cylinder (Figure 1A,B). Uncorrected Visual Acuity (UCVA) and Best Corrected Visual Acuity (BCVA) were evaluated for both distance and near vision for all patients. Additionally, intermediate vision acuity was determined with the best distance correction. Visual acuity evaluations were performed monocularly and binocularly using ETDRS optotypes calibrated to measure at distances of 3 m (distance), 0.65 m (intermediate), and 0.4 m (near) with a maximum contrast of 100%. Furthermore, to study the influence of contrast on distance visual acuity, measurements were taken with the best distance correction and a 20% contrast stimulus using the Colenbrander backlit screen.

Defocus curves were performed monocularly and binocularly over the patient’s best distance correction. There is a recommended dioptric range by consensus for performing defocus curves in the evaluation of EDOF lenses, which is at least from +1.50 D to −2.50 D [10]. In this study, the range for both lenses was from +1.50 D to −4 D in 0.5 D increments under photopic lighting conditions.

The discrimination index was measured using halometry. The evaluation of ocular light scatter was carried out using Halo v.1.0 software, developed by the Vision Sciences and Applications Laboratory at the University of Granada, Spain.

### 2.5. Questionnaires

The 2000 version of the validated Visual Functioning Questionnaire 25 (VFQ25) [11] was used to detect vision-related quality of life issues preoperatively and one month after surgery. This questionnaire is divided into three parts: questions related to vision and general health, questions related to difficulty with daily activities, and questions related to vision problems.

### 2.6. Statistical Analysis

Statistical analysis was performed using SPSS V17 software by separating the data into numerical and ordinal variables. For numerical variables, the Mann–Whitney U test, paired *t*-test for independent samples, and *t*-test for repeated measures were used. For ordinal variables, the Wilcoxon rank-sum test and paired *t*-test for repeated measures were used. Differences were considered significant at *p*-value < 0.05. For the randomization of the sample subjects, we used the Sealed Envelope Ltd. (London, UK) 2022 program, available at Sealed Envelope.

**Figure 2:**
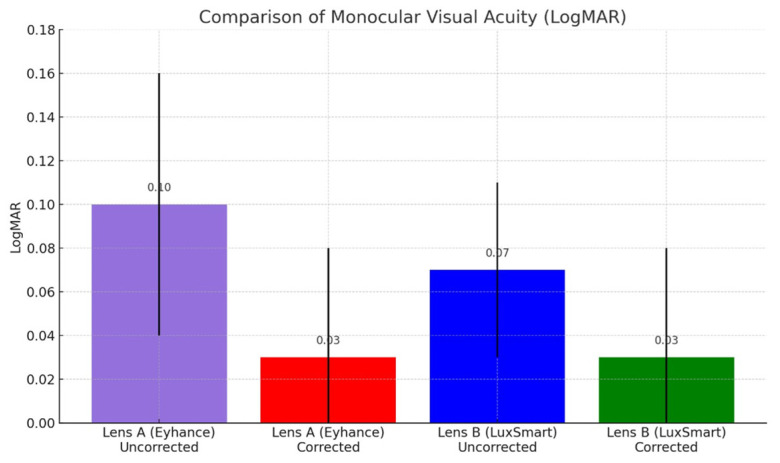
Comparison of distance visual acuity with and without monocular correction for Lens A (Eyhance) and Lens B (LuxSmart). The figure represents the mean monocular visual acuity values (LogMAR), and the error bars denote standard deviations. There were no statistically significant differences in uncorrected and corrected distance visual acuity between the two lenses.

Figure 1A,B and Figure 2 were generated with the assistance of ChatGPT (OpenAI, GPT-4) and subsequently validated by the authors.

## 3. Results

A total of 41 patients underwent cataract surgery and were included in the study. Of these, 22 patients received the Tecnis Eyhance™ intraocular lens (Lens A) and 19 patients received the LuxSmart™ intraocular lens (Lens B). For each patient, the eye selected for analysis was the first eye scheduled for surgery, independently of laterality. The mean age in the Lens A group was 75.77 ± 4.43 years (range: 66–84 years), while the Lens B group had a mean age of 71.79 ± 6.84 years (range: 60–86 years). Regarding sex distribution, 59.10% of the patients in group A were women, and 40.90% were men. In group B, 55.60% were women and 44.40% were men.

Descriptive statistics of the baseline parameters are presented in Table 1.

No statistically significant differences were found in the baseline characteristics of the samples, except for the preoperative pupil size, which was larger in Group B (2.85 ± 0.45 mm) than in Group A (2.37 ± 0.47 mm), *p* = 0.013.

### 3.1. Postoperative Refraction: Accuracy in Postoperative Refractive Error

Figure 1A,B show the refractive accuracy of the procedure by determining the spherical equivalent (top) and the refractive cylinder (bottom) at the last postoperative visit. The average postoperative spherical equivalent for Lens A (Eyhance) was −0.15 D ± 0.12 and for Lens B (LuxSmart) was −0.12 D ± 0.10, with no statistically significant difference (*p* = 0.705).

The average postoperative refractive cylinder for Lens A (Eyhance) was 0.091 D ± 0.12 and for Lens B (LuxSmart) was 0.11 D ± 0.10, also showing no statistically significant difference (*p* = 0.796).

### 3.2. Visual Acuity and Defocus Curves

As shown in Figure 2, the uncorrected postoperative monocular distance visual acuity (UDVA) was slightly better in the LuxSmart™ group (Lens B: 0.07 ± 0.02 LogMAR) compared to the Eyhance™ group (Lens A: 0.10 ± 0.06 LogMAR), although the difference did not reach statistical significance (*p* = 0.06). With best distance correction, both groups achieved equivalent monocular distance visual acuity (0.03 ± 0.05 LogMAR), with no statistically significant difference between them (*p* = 0.915).

Regarding postoperative binocular distance visual acuity with correction, LogMAR was 0.00 ± 0.02 for Lens A and 0.01 ± 0.03 for Lens B, with a *p*-value of 0.058.

The LogMAR data for postoperative monocular near visual acuity without optical correction were 0.30 ± 0.02 for Lens A and 0.32 ± 0.11 for Lens B, with a *p*-value of 0.88. With the best optical correction for near vision, the monocular values were 0.00 ± 0.02 for Lens A and 0.00 ± 0.00 for Lens B, with a *p*-value of 0.35. In a binocular situation with correction, both lenses presented a visual acuity value of 0.00 ± 0.00, with a *p*-value of 1.00. The intermediate visual acuity with distance vision correction was, monocularly, 0.17 ± 0.07 for Lens A and 0.16 ± 0.10 for Lens B, with a *p*-value of 0.95; in a binocular situation, it was 0.14 ± 0.07 for Lens A and 0.16 ± 0.08 for Lens B, with a *p*-value of 0.51.

A summary of LogMAR visual acuity is shown in Table 2.

In the analysis of postoperative visual acuity, both lenses showed comparable outcomes for most of the evaluated conditions, with no statistically significant differences observed.

For low-contrast visual acuity (20% contrast) under monocular conditions, the mean LogMAR VA was 0.22 ± 0.11 for Lens A and 0.26 ± 0.16 for Lens B (*p* = 0.49). Under binocular conditions, the corresponding values were 0.19 ± 0.09 for Lens A and 0.24 ± 0.15 for Lens B (*p* = 0.37), indicating no statistically significant differences.

**Figure 3:**
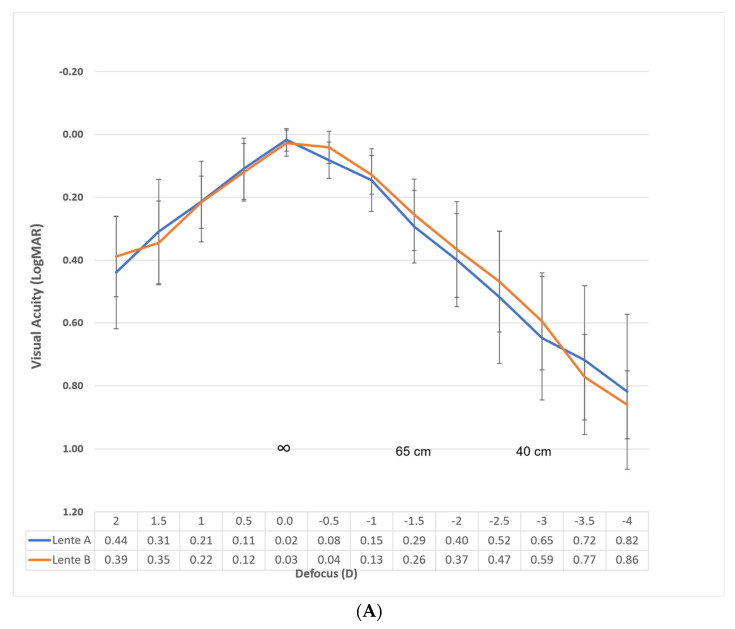

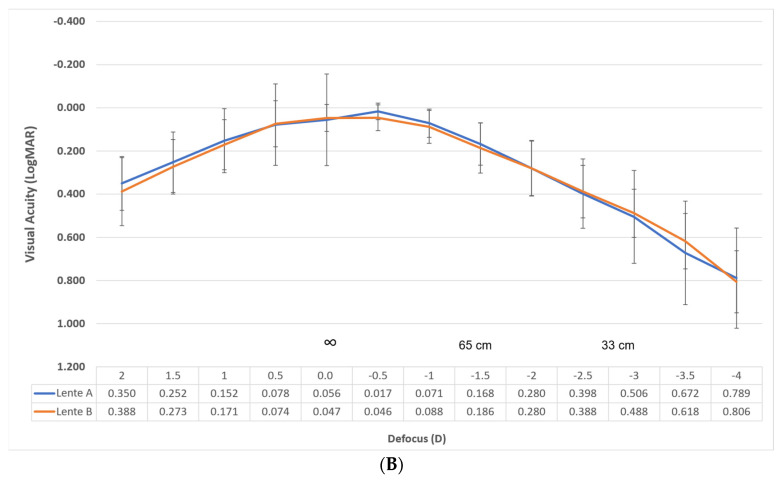
(**A**) Monocular defocus curve for Lens A (Eyhance) and Lens B (LuxSmart), with best distance correction. Visual acuity was evaluated at 0.5 diopter intervals across a range from +2 D to −4.00 D. Both lenses showed comparable performance at far and intermediate distances. The horizontal axis includes the vergence in diopters and the corresponding viewing distance in centimeters. Points represent mean visual acuity values, and error bars represent standard deviation. (**B**) Binocular defocus curve for Lens A (Eyhance) and Lens B (LuxSmart), with best distance correction measured under the same conditions as the monocular curve.

Figure 3A shows the monocular defocus curve with the best distance correction for evaluating the performance of each lens. Figure 3B shows the defocus curve in binocular conditions under the same circumstances for evaluating the success of the procedure.

Specifically, the binocular Corrected Distance Visual Acuity (CDVA) for Lens A was 0.07, 0.14, and 0.29 logMAR at −1.00, −1.50, and −2.00 D, respectively. For Lens B, within the same defocus range, the CDVA was 0.07, 0.16, and 0.28. These vergences correspond to distances of 1 m, 66 cm, and 50 cm, respectively. Considering the depth of focus as the range of lens powers that achieve a mean visual acuity of LogMAR 0.2 or better, the overall value is approximately 2.75 D. If we consider only from 0.00 D vergence, our results reveal a value of approximately 1.75 D for both lenses.

### 3.3. Light Discrimination Index

As shown in Table 3, patients in the Lens B group presented slightly lower preoperative light discrimination, as indicated by a Light Discrimination Index (LDI) of 0.69 ± 0.17, compared to 0.75 ± 0.15 in the Lens A group; however, this difference did not reach statistical significance (*p* = 0.15).

After surgery, both groups showed improved discrimination of light stimuli, reaching very similar postoperative LDI values: 0.82 ± 0.13 for Lens A and 0.83 ± 0.13 for Lens B. Again, the difference was not statistically significant (*p* = 0.54), indicating that both lenses achieved comparable outcomes in terms of reducing perceived halo intensity and improving visual clarity under glare conditions.

### 3.4. Quality of Life Questionnaire

**Figure 4:**
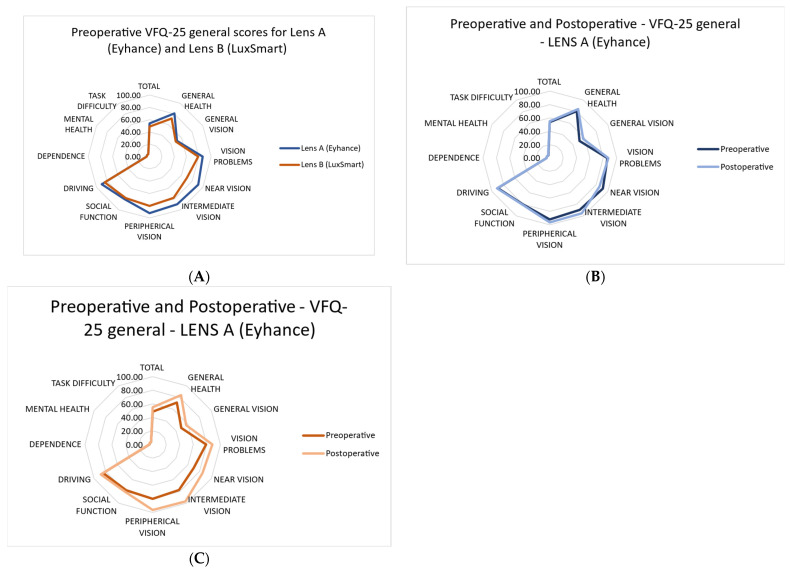
(**A**) Preoperative NEI VFQ-25 general scores for Lens A (Eyhance) and Lens B (LuxSmart) groups. Each axis represents a specific domain of vision-related quality of life. Higher values (closer to the outer edge) indicate better perceived visual function. The blue line (Lens A–Eyhance) and the orange line (Lens B–LuxSmart) reflect the mean scores reported by each group before surgery. (**B**) Preoperative and postoperative NEI VFQ-25 scores for patients implanted with Lens A (Eyhance). The dark blue line represents preoperative scores, while the light blue line shows postoperative scores, demonstrating a mild general improvement after cataract surgery. (**C**) Preoperative and postoperative NEI VFQ-25 scores for patients implanted with Lens B (LuxSmart). The dark brown line represents preoperative scores, while the light brown line shows postoperative scores, illustrating overall improvement following cataract surgery.

As shown in Figure 4, patients who received Lens A (Eyhance™) appeared to report a more favorable perception of vision-related aspects prior to surgery compared to those who received Lens B (LuxSmart™).

Comparing preoperative and postoperative values for each lens separately showed a general improvement in the performance of daily activities after cataract surgery.

Detailed descriptive statistics for each question in the questionnaire are presented in Table 4.

There are no statistically significant differences between patient responses to the vision-related questions in the provided questionnaire, as indicated by the following *p*-values: P5 = 0.28, P6 = 0.93, P7 = 0.42, P8 = 1.0, P9 = 1.0, P11 = 1.0, and P14 = 0.46. The results show a high level of satisfaction with the vision obtained after surgery.

## 4. Discussion

This study is the first to evaluate the clinical performance of the LuxSmart™ intraocular lens, a purely refractive extended depth-of-focus (EDOF) IOL, in patients with early-stage age-related macular degeneration (AMD). The use of presbyopia-correcting IOLs in patients with AMD has traditionally been controversial due to concerns that multifocal and diffractive optics may reduce contrast sensitivity and impair visual quality, especially under low-light conditions. These effects may further compromise the already diminished retinal function in AMD patients. However, non-diffractive EDOF lenses, such as LuxSmart™, employ purely refractive optics designed to extend depth of focus without splitting light into multiple focal points. This design avoids light loss and contrast degradation typically associated with diffractive IOLs. The results were compared to those obtained with the Tecnis Eyhance™ monofocal plus IOL, which served as a control group.

To date, only one other published study has explored the use of a refractive EDOF IOL—the AcrySof IQ Vivity™ (Alcon Laboratories, Fort Worth, TX, USA)—in patients with AMD at various stages. In that study, seven eyes presented early-stage AMD, and the visual outcomes were comparable to those in our sample; specifically, they reported a corrected distance visual acuity (CDVA) of 0.00 ± 0.09 LogMAR and a corrected distance near visual acuity (CDNVA) equivalent to N7 on the Times New Roman scale (approximately LogMAR 0.4) [12].

Extended depth of focus (EDOF) technology is applied to intraocular lenses (IOLs) for presbyopia correction with the aim of providing good visual results over a wide range of distances.

The results obtained show that the purely refractive EDOF IOL provides excellent results in terms of refractive accuracy, demonstrating that the surgery achieves precise correction of both the spherical component and corneal astigmatism.

In the evaluation conducted one month after surgery, all eyes (100%) presented a spherical equivalent of ±0.50 and an uncorrected distance visual acuity (UDVA) of LogMAR 0.075 or better, with a mean of LogMAR 0.07 ± 0.02. These results are largely consistent with previous clinical studies, which have demonstrated the excellent refractive accuracy of this lens after implantation, such as the study by Campos et al. [5], in which the lens was bilaterally implanted in 12 patients and evaluated at 3 months, obtaining a mean spherical equivalent of −0.08 ± 0.60 D, with 91.7% of the eyes within ±0.50 D and a binocular UDVA of 0.02 ± 0.11.

Similarly, Tahmaz et al. [13], in a study of 28 patients bilaterally implanted and evaluated at 3 months, found that 96% and 98% of the eyes were within ±0.50 D and ±1.00 D, respectively, and presented a binocular UDVA of 0.04 ± 0.06.

Nowrouzi et al. [14], in an evaluation (conducted at 24 weeks) of 25 eyes unilaterally implanted, reported a mean spherical equivalent of −0.25 ± 0.50 D, with 80% and 92% of the eyes within ±0.50 D and ±1.00 D, respectively, and a monocular UDVA of 20/25 (LogMAR 0.1) or better in 100% of the eyes.

More recently, Ruiz Mesa et al. [15], evaluating the toric platform of the lens in 44 eyes of 22 patients, found that 90.45% and 100% of the operated eyes were within ±0.50 D and ±1.00 D, respectively, with a mean postoperative spherical equivalent of −0.02 ± 0.26 D. Regarding astigmatism, 93.18% and 100% of the eyes were within ±0.50 D and ±1.00 D, respectively, with a mean value of −0.17 ± 0.29 D. In this case, the binocular UDVA was 0.00 ± 0.06.

The results of all the evaluated studies demonstrate the refractive accuracy of the lens calculation and excellent performance in uncorrected distance vision, whether mono or binocular.

This lens also provides high values of binocular distance visual acuity with correction (CDVA), with mean values in our sample of 0.03 ± 0.00. Additionally, 100% of the patients presented a cumulative binocular CDVA of 20/21 or better.

Similar results have been reported in previous studies. Campos et al. found mean values of −0.08 ± 0.04 logMAR for binocular CDVA. Tahmaz et al. reported mean values of 0.00 ± 0.05 logMAR for binocular CDVA. Ruiz Mesa et al. found mean values of binocular distance visual acuity with correction (CDVA) of −0.02 ± 0.06 logMAR. Additionally, 95.45% of the patients presented a cumulative binocular CDVA of 20/25 or better.

In the intermediate range of distances (evaluated at 65 cm), monocular intermediate visual acuity with distance correction (DCIVA) with the LuxSmart IOL was 0.16 LogMAR; similarly, the monocular near visual acuity with distance correction (DCNVA) at 40 cm was 0.38 LogMAR.

Our results are in line with those published by Nowrouzi et al., who, in the intermediate range of distances (evaluated at 60 cm), found that the intermediate visual acuity with distance correction (DCIVA) with the LuxSmart IOL was 0.2 LogMAR and the near visual acuity with distance correction (DCNVA) at 40 cm was 0.38 LogMAR. It is worth noting that in this study, the results were evaluated in younger patients with unilateral posterior subcapsular cataracts, with a mean age of 54 years compared to the 71-year mean age of patients in our sample.

Regarding binocular intermediate vision, the results obtained were also favorable: the mean binocular DCIVA was 0.15 ± 0.10 logMAR. Our results are slightly lower than those of Ruiz Mesa et al., who reported a mean value of 0.08 ± 0.07 logMAR. The values reported by Tahmaz et al. were more comparable to ours, despite measuring intermediate visual acuity at 80 cm, obtaining mean values of 0.12 ± 0.11 logMAR for binocular DCIVA.

The results for near vision were lower than those obtained for distance and intermediate vision. The mean binocular near visual acuity with correction (CDNVA) was 0.37 ± 0.10 logMAR.

These findings are consistent with previous studies, such as that of Campos et al., who reported a mean value of 0.38 ± 0.14 logMAR for binocular UNVA, and Ruiz Mesa et al., who found a mean value of 0.26 ± 0.09 logMAR for binocular CDNVA.

Regarding low contrast visual acuity, the binocular LogMAR VA data for the LuxSmart lens of 0.24 ± 0.15 are in line with those reported by Gundersen et al. [16], who evaluated low contrast visual acuity at 10% for the EDOF Symfony^®^ lens (J&J Vision, Inc., Santa Ana, CA, USA) using the M&S Technologies Clinical Trial Suite test (Niles, IL, USA) and obtained values close to LogMAR 0.3. These values fall within the range of functional vision used in the evaluations of vision ranges in defocus curves [17].

The visual acuity results correlate with the defocus curves, represented in Figure 3A,B, where good visual acuity is observed for distance and intermediate distances (up to 50 cm), while visual acuity decreases at closer distances (40 or 33 cm).

Tahmaz et al. evaluated the monocular depth of focus of this lens, obtaining a value of 1.60 D at 20/32 (0.2 LogMAR), derived from the defocus curve. Nowrouzi et al. obtained the same results. Our findings were superior and more comparable to those reported by Campos et al. and Ruiz Mesa et al., who reported a binocular depth of focus of approximately 2/2.25 D. In our case, the range of vision above 0.2 LogMAR was 2.75 D, and if we consider only from 0.00 D vergence, it was 1.75 D.

Considering these results, it can be observed that the LuxSmart lens performs similarly in patients with early-stage AMD as in patients without it. Additionally, the non-diffractive EDOF technology is safe and provides a satisfactory range of vision in patients with early-stage AMD, as evidenced by both our results and those of Thananjeyan et al. [12].

Regarding the control group, we found excellent performance of the Eyhance lens, with no statistically significant differences in any aspect compared to the LuxSmart lens, except in the monocular defocus curve, in the range of −0.50 diopters, where the LogMAR VA of patients with LuxSmart was 0.02 ± 0.00 and with Eyhance was 0.08 ± 0.00, *p* = 0.02, without considering this difference clinically relevant. Our results with the Eyhance lens are also comparable to those of other authors, although slightly inferior, such as Giglio et al., who presented a binocular CDVA of −0.07 ±0.05, a DCIVA of 0.13 ± 0.11, and a DCNVA of 0.23 ± 0.11 at three months of follow-up [18], or Auffarth et al., who presented a binocular CDVA of −0.06 ± 0.09 and a DCIVA of 0.09 ± 0.11 LogMAR at six months of follow-up [19]. Evaluating the results of our sample with early-stage AMD under the same conditions, we obtained a binocular CDVA of 0.00 ± 0.20, a DCIVA of 0.14 ± 0.10, and a DCNVA of 0.38 ± 0.10.

Considering the results, both lenses are comparable, even with slightly inferior performance of the Eyhance lens in our sample compared to other authors.

Regarding the halometry results, in our case, the measurement of the Light Discrimination Index (LDI) did not show statistically significant differences between the two lenses, presenting postoperative values close to unity, which implies lower light scatter, with LuxSmart lens at 0.82 ± 0.13 and Eyhance lens at 0.83 ± 0.13, *p* = 0.54. Our results are inferior to those published by Baoud et al. [20], who found a binocular halometry value of 0.94 ± 0.08 for the Eyhance lens, but very similar to those reported by Garzón et al. [21], who obtained a value of 0.819 ± 0.117 using the same test, although their samples did not include patients with AMD.

In the quality-of-life results reported by patients via the VFQ-25 questionnaire, an improvement in the performance of daily activities after cataract surgery was observed. However, no significant changes were found in aspects such as task difficulty, mental health, dependency, driving, or social function after surgery, which may be attributed to the preoperative visual acuity data not being low enough to affect these aspects.

The results obtained from the VFQ-25 questionnaire indicate that 89% of patients were quite satisfied (47%) or very satisfied (42%) with their vision after surgery. Table 4 presents the frequency of responses to questions related to the difficulty of performing daily activities, as measured by the VFQ-25.

It is important to note that more than 70% of patients did not report difficulties performing near-vision activities, such as reading newspaper texts (P5, 73.7%) and performing manual tasks (P6, 94.8%). Regarding questions related to intermediate vision (P8 and P11), the results showed that patients had little or no difficulty in 99% of cases for P8 and 100% of cases for P11. Questions related to distance vision (P7 and P14) and mesopic vision were estimated by evaluating the difficulty in activities such as stepping off a curb or stairs at night (P9). The results for both distance and night vision showed that 100% of patients had little or no difficulty performing the evaluated activities.

Our findings fully align with those reported by Ruiz Mesa et al., based on the Catquest-9SF questionnaire, in their series of patients, indicating that 90.9% of patients were quite satisfied (11 of 22) or very satisfied (9 of 22) with their vision after surgery, and more than 70% of patients did not report difficulties in near-vision activities, such as reading newspaper texts (77%), identifying prices during shopping, and performing manual tasks (72.8%). Campos et al. also used the Catquest-9SF questionnaire in their sample. In their study, most patients reported high levels of satisfaction with their daily activities, particularly reading newspapers, identifying prices in stores, and using the computer. Additionally, 83.3% of their patients were very satisfied with their vision after surgery.

The results of the VFQ-25 questionnaire showed an improvement in daily activities after surgery. Despite the underlying pathology, more than 70% of patients did not report difficulties in near and intermediate vision tasks, and 89% declared themselves quite or very satisfied with their postoperative vision. These findings reinforce the functional value of surgery in this population, even in the presence of AMD. Our results are consistent with previous studies, which also reported high levels of satisfaction and minimal difficulty with visual tasks after surgery. Based on the Catquest-9SF questionnaire, Ruiz Mesa et al. reported that 90.9% of patients were quite satisfied or very satisfied with their vision after surgery, and more than 70% of patients did not report difficulties performing near vision activities. 

### Limitations

The short patient follow-up period is a limitation of the study; however, several studies suggest that refractive stability after cataract surgery can be achieved as early as two weeks [22,23]. The worse results compared with other studies using the control lens are due to patients having slightly smaller pupils, and the use of the femtosecond laser, which may not be widely available due to its high cost. This study was not blinded, and neither patients nor evaluators were masked to the type of intraocular lens implanted. This lack of blinding may have introduced some degree of bias, particularly in subjective outcome measures such as visual satisfaction and quality-of-life questionnaires. Although objective metrics such as visual acuity and refractive outcomes were unlikely to be influenced, caution should be exercised when interpreting results related to patient-reported outcomes.

## 5. Conclusions

Our findings indicate that both the purely refractive EDOF technology LuxSmart™ intraocular lens (IOL) and the monofocal plus Eyhance™ lens provide good refractive, optical, and visual quality at different distances, with a high level of satisfaction among implanted patients. These lenses can be considered a safe option for patients with cataracts and early-stage AMD who seek independence from glasses for distance and intermediate vision, thanks to the extended focus provided by EDOF and monofocal plus technologies.

## 6. Patents

There are no patents resulting from the work reported in this manuscript.

## Figures and Tables

**Table 1:** Baseline characteristics and potential factors influencing visual outcomes. Comparison between Lens A (Eyhance™) and Lens B (LuxSmart™).

Preop	Group A (Eyhance)	Group B (Luxsmart)
**Sample size (N)**	22	19
**Sex (F/M)%**	59.10/40.90	55.60/44.40
**Age (years)**	75.77 ± 4.43	71.79 ± 6.84
	(66–84)	(60–86)
**Cataract type (LOCS III)**	3.54 ± 1.14	2.94 ± 1.13
	(1–5)	(1–5)
**Lens power (D)**	21.54 ± 2.00	20.74 ± 2.18
	(19–25)	(16–26,5)
**Pupil size (mm)**	2.37 ± 0.47	2.85 ± 0.45
	(2.04–3.78)	(2.06–4.29)
**Aberrometry—Z40 (µm)**	0.42 ± 0.17	0.38 ± 0.12
	(0.122–0.899)	(0.151–0.664)
**Aberrometry—HOA (µm)**	0.24 ± 0.08	0.20 ± 0.07
	(0.128–0.451)	(0.07–0.395)

**Table 2:** Summary of LogMAR visual acuity.

Condition	Lens A (LogMAR)	Lens B (LogMAR)	*p*-Value
Uncorrected distance vision (monocular)	0.10 ± 0.06	0.07 ± 0.02	0.06
Corrected distance vision (monocular)	0.03 ± 0.05	0.03 ± 0.05	0.915
Corrected distance vision (binocular)	0.00 ± 0.02	0.01 ± 0.03	0.058
Uncorrected near vision (monocular)	0.30 ± 0.02	0.32 ± 0.11	0.88
Corrected near vision (monocular)	0.00 ± 0.02	0.00 ± 0.00	0.35
Corrected near vision (binocular)	0.00 ± 0.00	0.00 ± 0.00	1.0
Intermediate vision with distance correction (monocular)	0.17 ± 0.07	0.16 ± 0.10	0.95
Intermediate vision with distance correction (binocular)	0.14 ± 0.07	0.16 ± 0.08	0.51

**Table 3:** Results of the Halo V1 test pre and postoperatively.

		Halometry		
Halo V.1.0	Sample Size, N (Lens A/Lens B)	Group A (Eyhance) (X ± σ)	Group B (Luxsmart) (X ± σ)	*p*-Value
**IDL PREOP**	**22/19**	0.75 ± 0.15	0.69 ± 0.17	0.15
**IDL POSTOP**	**22/19**	0.82 ± 0.13	0.83 ± 0.13	0.54

**Table 4:** Summary of postoperative results reported by patients in the VFQ-25 questionnaire (Part 2, which evaluates the difficulty encountered when performing daily life tasks).

VFQ-25 LuxSmart					
Percentage frequencies	R1	R2	R3	R4	R5
P5- How much difficulty do you have reading normal print in newspapers?	42.1	31.6	26.3	0	0
P6. How much difficulty do you have doing work or hobbies that require good near vision, such as cooking, fixing things around the house, or using tools?	52.7	42.1	5.2	0	0
P7. Because of your eyesight, how much difficulty do you have seeing traffic signs or store names?	89.5	10.5	0	0	0
P8. How much difficulty do you have finding something on a crowded shelf?	84.2	15.8	0	0	0
P9. Because of your eyesight, how much difficulty do you have going down steps, stairs, or curbs in dim light or at night?	78.9	21.1	0	0	0
P11. Because of your eyesight, how much difficulty do you have seeing how people react to what you say?	100	0	0	0	0
P14. Because of your eyesight, how much difficulty do you have going to see movies, plays, or sporting events?	94.7	5.3	0	0	0
R1 = No difficulty; R2 = A little difficulty; R3 = Moderate difficulty; R4 = Extreme difficulty; R5 = Stopped doing this because of eyesight					
**VFQ-25 Eyhance**					
Percentage frequencies (%)	R1	R2	R3	R4	R5
P5- How much difficulty do you have reading normal print in newspapers?	77.3	13.6	9.1	0	0
P6. How much difficulty do you have doing work or hobbies that require good near vision, such as cooking, fixing things around the house, or using tools?	59.1	31.8	9.1	0	0
P7. Because of your eyesight, how much difficulty do you have seeing traffic signs or store names?	77.3	22.7	0	0	0
P8. How much difficulty do you have finding something on a crowded shelf?	81.8	13.6	4.9	0	0
P9. Because of your eyesight, how much difficulty do you have going down steps, stairs, or curbs in dim light or at night?	77.3	22.7	0	0	0
P11. Because of your eyesight, how much difficulty do you have seeing how people react to what you say?	100	0	0	0	0
P14. Because of your eyesight, how much difficulty do you have going to see movies, plays, or sporting events?	100	0	0	0	0
R1 = No difficulty; R2 = A little difficulty; R3 = Moderate difficulty; R4 = Extreme difficulty; R5 = Stopped doing this because of eyesight					

## Data Availability

The data presented in this study are not publicly available due to patient privacy and ethical restrictions. Data may be made available upon reasonable request to the corresponding author, subject to approval by the institutional ethics committee.

## Supplementary Materials

The Halo Software User Manual can be downloaded at: https://digibug.ugr.es/handle/10481/5477?show=full (accessed on 31 January 2025).
